# Does partnership diversity in intersectoral policymaking matter for health promoting intervention packages’ composition? A multiple-case study in the Netherlands

**DOI:** 10.1093/heapro/daaa083

**Published:** 2020-08-20

**Authors:** K M Grêaux, N K de Vries, K M H H Bessems, J Harting, P van Assema

**Affiliations:** 1 Department of Health Promotion, NUTRIM School of Nutrition and Translational Research in Metabolism, Maastricht University Medical Centre, PO Box 616, MD Maastricht 6200, The Netherlands; 2 Department of Public Health, Academic Medical Center, University of Amsterdam, PO Box 22660, DD Amsterdam 1100, The Netherlands

**Keywords:** intersectoral partnerships, healthy public policy, implementation, intervention

## Abstract

Intersectoral policymaking to improve public health includes integrated health promotion (HP) intervention packages that address a variety of health behavior determinants. The involvement of different partners is assumed to be necessary to implement such integrated packages. We examined how partnership diversity was associated with the composition of intervention packages implemented in Dutch municipalities. In a longitudinal multiple-case study (2012–14), we collected questionnaire data among 31 project leaders and 152 intervention implementers in 31 (alliances of) municipalities. Package composition was assessed in terms of intervention strategies, implementation settings and targeted behavioral determinants. Partnership diversity during the adoption and implementation phases was assessed in terms of the actors and sectors, as well as private partners and citizens involved. The association between partnership diversity and package composition was examined using crosstabs. Almost all packages integrated multiple strategies, but mostly education, facilitation and case finding, in multiple, but mostly health and public settings, such as schools. The packages targeted diverse behavioral determinants, although mainly personal and social environmental factors. A variety of partners from multiple sectors was involved, during both adoption and implementation of the packages. However, partners from the health, welfare and education sectors were mostly involved. More partnership diversity, especially during implementation, was associated with more integrated intervention packages. In intersectoral policymaking, investment in diversely composed partnerships seems worthwhile for implementing integrated intervention packages*.* However, investments in other conditions, like framing health issues and network management, are also needed to make environmental determinants of health behavior the object of HP.

## BACKGROUND

Countries worldwide are facing high economic and social burdens due to a pandemic of non-communicable diseases (Mayer-Foulkes, 2011; WHO, 2018). These diseases are primarily caused by unhealthy lifestyle behaviors (e.g. poor diet, sedentary behavior, and alcohol and drugs abuse) (Mayer-Foulkes, 2011; WHO, 2018) and have a ‘wicked’ character (Rittel and Webber, 1973; Signal *et al.*, 2013). This wicked character refers, among other things, to the complex interactions between personal (e.g. attitudes, skills, motivation) and environmental behavioral determinants (e.g. social, physical, economic and political) (Australian Government, 2007; Sallis *et al.*, 2008). To effectively promote health, experts therefore often advocate intersectoral health policy (Smedley and Syme, 2000; Kickbusch and Gleicher, 2012). In essence, such a policy integrates complementary policy strategies from different policy sectors as to achieve the coordinated action needed to address the variety of determinants underlying health and health behavior (Smedley and Syme, 2000).

In many countries, including the Netherlands, it is local governments who are responsible for intersectoral health policymaking at the local level (Atkinson *et al.*, 2000; Storm *et al.*, 2011). Preferably, this would result in the implementation of ‘integrated health promotion (HP) packages’ (Australian Government, 2007; Hunter, 2009). Here, ‘integrated’ means that these packages are composed of complementary intervention strategies (e.g. education and regulation), are situated in a variety of local settings (e.g. schools and public places) and are targeted at both personal and various types of environmental behavioral determinants (Jackson *et al.*, 2006; Storm *et al.*, 2011; Bloch *et al.*, 2014). Such packages are assumed to require diverse partnerships (Jackson *et al.*, 2006; Kickbusch *et al.*, 2008; Bloch *et al.*, 2014), as to ensure the necessary collaborative action of a variety of partners that goes beyond the health sector (Clavier *et al.*, 2012). As part of intersectoral policymaking, this ‘partnership diversity’ is expected to be needed both when the decisions to adopt interventions are made (i.e. the adoption phase) and when the target population is actually exposed to interventions (i.e. the implementation phase) (Saxe *et al.*, 1997; Pentz, 2004; Valente *et al.*, 2007).

Take, for example, an integrated HP package to reduce alcohol abuse among young people. Such a package may include regulatory measures, such as age limits for buying alcohol, which should be adopted by councilors, and implemented by both public security officials and alcohol distributors. The same HP package may include educational strategies, to inform young people about the harmfulness of alcohol, which should be adopted by both school directors and parents’ councils, and implemented by teachers. This simplified example illustrates the common idea that both partnership diversity and integrated intervention packages are conditional for intersectoral health policy.

Although the relevance of intersectoral health policymaking has been widely stressed, in practice it remains difficult (Holt *et al.*, 2017). For example, involving a variety of partners as well as making intersectoral partnerships work have appeared to be very complex (Shankardass *et al.*, 2012; Corbin *et al.*, 2018). It often requires substantial time and managerial investments to start and maintain intersectoral collaborations (Axelsson and Axelsson, 2006; Clavier *et al.*, 2012; Koelen *et al.*, 2012; Peters *et al.*, 2017a). This is due to, for instance, the challenge of identifying the right partners, existing cultural and structural barriers, and differences in the partners’ perceptions of goals, procedures and success (Edvardsson *et al.*, 2012; Koelen *et al.*, 2012; Varda and Retrum, 2012). In search of how to deal with these barriers, review studies found that positive partnership processes tend to include various core elements, such as developing a shared mission, incorporating leadership, arranging technical assistance and support, monitoring communication, building trust, balancing roles and structures depending upon mission, securing financial resources, making results matter, and evaluation and feedback for improvement (Roussos and Fawcett, 2000; Corbin *et al.*, 2018).

Despite the required investments, there is only limited empirical support for the assumed positive relationship between partnership diversity and the realization of integrated HP packages. One qualitative multiple-case study found that intersectoral health networks may indeed support local health action addressing environmental determinants (Clavier *et al.*, 2012). However, two other multiple-case studies on intersectoral programs found that, despite the program’s intentions, even in the presence of a variety of partners establishing integrated HP packages that address a variety of environmental determinants is not self-evident (Peters *et al.*, 2016; Holt *et al.*, 2017). These contrasting findings may illustrate the conclusion of a scoping review, that only a minority of the evaluated government-centered intersectoral initiatives had managed to address structural determinants of health (Shankardass *et al.*, 2012).

As a limitation, the scoping review found that most of the included studies did not provide much documentation on the complex multi-actor processes that are intrinsic to intersectoral approaches (Shankardass *et al.*, 2012). Two other reviews observed a paucity of research examining the relationship between intersectoral partnership processes and objective outcome measures as a result of methodological challenges (Roussos and Fawcett, 2000; Corbin *et al.*, 2018). For example, as evidence of a partnership’s impact, such as health policies developed and HP programs implemented, is difficult to collect, quantitative outcomes like these were not assessed in the evaluation of partnerships in the WHO Healthy Cities network (Lipp *et al.*, 2013). Another limitation of the available evidence is that the scarce studies that did examine the relationship between partnership processes and outcomes did not differentiate between different phases in the process of realizing HP interventions. Therefore, it is currently unclear when partnership diversity would be most important: when adoption decisions are made about the composition of the HP packages or when such packages are implemented in practice (Varda and Retrum, 2012; Kleij *et al.*, 2015). We argue that the investments required, the difficulties engaged and the uncertainty of the evidence available, warrant further—and also quantitative—study of the premise that partnership diversity contributes to the implementation of integrated HP packages.

To determine whether it is indeed worth continuing the challenging ‘endeavor’ of involving diverse partners in local intersectoral health policymaking, we aimed to clarify the following research question: ‘Does partnership diversity matter for the composition of intervention packages implemented?’ To answer this question, we used quantitative data from health policy programs in Dutch municipalities to assess: (i) the composition of the HP packages in terms of the strategies, settings, and targeted behavioral determinants of the interventions, (ii) partnership diversity during the adoption and implementation phases, in terms of the partners and sectors involved, as well as the involvement of private partners and citizens and (iii) the association between partnership diversity during both the adoption and implementation phase and the composition of the packages actually implemented.

## METHODS

### Setting

The Gezonde Slagkracht program (Decisive Action for Health program; 2009–15) was a program of the Dutch Ministry of Health, Welfare and Sport (ZonMw, 2009). The program gave municipalities the opportunity to experiment with intersectoral health policymaking over a period of 24 − 48 months on one or more of the following themes: nutrition, physical activity, alcohol, drugs and smoking. The program could be characterized as a procedural program (Clavier *et al.*, 2012), i.e. a governmental tool that determines guidelines and provides resources, but no specific prescriptions on the content. Municipalities could apply for participation in the program. The requirements included the appointment of a municipal project leader who had to take a coordinating role in both the establishment of local partnerships and the implementation of integrated HP packages. Partnerships were expected to involve a wide range of partners, from the health sector as well as the non-health sectors, and also private partners and citizens. HP packages were expected to include different types of HP interventions in various local settings as to address both personal and environmental health behavior determinants. The ministerial program provided financial support, ranging from 100 000 to 250 000 euro per project, to cover the appointment of the project leader and the implementation of HP packages. Additional professionals support offered by the ministerial program included workshops on national regulations affecting public health policy, interactive policy development, implementing evidence-based interventions and policy continuation.

### Study design

Data were collected as part of a longitudinal multiple-case study among 33 out of the 34 municipalities or alliances of municipalities that participated in the program (referred to below as projects), as one municipality prematurely ended its participation. Data collection took place at different points in time during the 2012–14 period. Each project was approached twice a year and invited to complete two data collection instruments. However, due to the variety in the starting and end dates of projects, and taking into account the different rates of progress of the projects (e.g. delay in decision making or implementation), the number of times that data were collected differed between projects.

The study was exempt from ethical review according to prevailing Dutch standards because the study was conducted among adults, considered to be low risk, participation was voluntary and completion of the questionnaires was considered to be equivalent to assent (CCoRIH, 2014).

### Data collection instruments

To capture the composition of the packages and partnership diversity, two questionnaires were developed: one for project leaders and one for each person with the prime responsibility for the implementation of at least one intervention (i.e. prime implementers). Project leaders who were also prime implementers received both questionnaires. After pretesting, the questionnaires were sent in printed form by postal mail and as Word documents by e-mail, and could be completed handwritten or electronically.

The 33 *project leaders* were invited to fill in the questionnaire in April of 2012, 2013 and 2014. Questions relevant for the current study addressed: (i) characteristics of the project leader (years of working experience, name of organization), (ii) confirmation of the project’s health theme(s) and target group(s) as derived from the project proposal, (iii) partners involved in the decisions to adopt interventions over the previous year, (iv) the number and names of interventions implemented over the previous year and (v) the prime implementers of these interventions.

If project leaders did not return the questionnaire, they were reminded three times: twice by e-mail and once by phone. When project leaders returned an incomplete questionnaire, they were approached by phone to clarify or complete their answers. Project leaders were also asked to send an announcement e-mail to the indicated prime implementers in their project including a request to participate in the study. In this phase, two projects refused permission to approach prime implementers (e.g. for reasons of time investment) and were therefore excluded from data analyses.

One hundred and ninety-five *prime implementers* for whom correct contact information was available were asked by the research team to complete the questionnaire for each of their interventions separately. A first set of open questions addressed the characteristics of the prime implementer (e.g. years of work experience, name of their organization). Regarding the intervention, a second set of open questions asked the prime implementer to concisely describe the intervention’s aim, content and implementation setting. Next, a pre-structured question asked to tick off the behavioral determinants that the intervention addressed (i.e. personal determinants and/or determinants in the social, physical, political and/or economic environment). An explanation for each of these categories was provided in the questionnaire. Finally, an open question asked the prime implementers to list which other partners were involved during implementation.

When implementers did not return the questionnaire, reminder e-mails were sent twice. If a non-responding implementer was responsible for more than one intervention, they were also reminded by telephone. We encouraged implementers to ask colleagues to help with filling in the questionnaire(s). We also offered to help by filling in the questionnaire(s) together during a phone call. A total of 38 out of the 85 (44.7%) implementers made use of the latter option, especially those who had to report more than one intervention.

### Data processing

To prepare for data analysis, we first classified the answers to the open questions about the intervention and the partners involved. The strategies employed in an intervention were retrieved from the description of its aim and content, and categorized into (Eldredge-Bartholomew *et al.*, 2016): education (e.g. school learning module), regulation (e.g. legislation regarding the sale of alcohol products in sports cafeteria’s during youth activities), facilitation (e.g. environmental or organizational changes such as new play gardens, supplying sports activities or materials), citizen participation (e.g. citizens organizing a walking event) and case finding (e.g. spotting drunk youngsters in nightlife). The setting(s) were categorized into (Poland *et al.*, 2000): school or preschool, sports facility, outdoor public site (e.g. playgrounds, nature areas), home (including websites to be consulted at home), health or welfare building (e.g. hospital, welfare organization, addiction center), public building (e.g. library, community centers) and commercial building (e.g. supermarkets, bars, restaurants). The partners involved were classified into the following sectors: municipal government organization (e.g. policy employees from various departments), education, sports, welfare, public health (e.g. regional public health organizations), primary care (e.g. addiction institutes), secondary care (e.g. hospitals), cultural/recreational/social (e.g. community centers), transportation and safety (e.g. police), bars and restaurants, and other businesses (e.g. retail stores, supermarkets). In addition to the sectors, partners were also categorized as private (for-profit market organizations) or non-profit, and as citizen group or not. The types of behavioral determinants targeted by the interventions were primarily derived from the pre-structured question on this topic. If the description of the aim and content of the intervention revealed that other determinants were being addressed, this information was merged with that of the pre-structured question.

### Data analysis

At *intervention level*, descriptive analyses were used to describe the characteristics of the interventions (themes, target groups, strategies, settings and targeted behavioral determinants) and partner involvement and partnership diversity during intervention implementation (sectors involved, number of different partners and sectors involved, and involvement of private and citizen partners).

To enable analyses of the *intervention packages*, we aggregated the data that were collected at the intervention level to the level of projects. Descriptive analysis was also used to describe the characteristics of the intervention packages, the composition of the packages in terms of the number of interventions, and the numbers of different strategies, settings and targeted behavioral determinants, as well as partnership diversity during the phases of adoption and implementation. The association between partnership diversity during the adoption and implementation phases and the composition of the intervention packages was assessed using crosstabs, crossing the numbers of different strategies, settings, and targeted behavioral determinants with the numbers of different partners and sectors involved and the percentage of projects with private partners and citizen partners. Additionally, the numbers of different strategies, settings and targeted behavioral determinants were crossed with the total number of interventions per project. We used IBM SPSS Statistics for Windows (Version 21.0, IBM Corp., Armonk, NY) for data analyses.

## RESULTS

### Response

Depending on the start and end date of their projects, the project leaders either returned one (*n* = 1), two (*n* = 16) or all three annual questionnaires (*n* = 14). In the 31 projects, 209 prime implementers implemented 488 interventions. The 195 implementers for whom we possessed correct contact information were responsible for the implementation of 423 interventions. Data on 315 of these interventions (74.0%) were returned by 158 of the invited implementers (response rate 81.0%). Thirteen questionnaires returned by six implementers were excluded from data analysis since <20% of the questionnaire items had been completed. In the end, data on 302 of the initial 488 interventions (61.9%) from 152 of the initial 209 implementers (72.7%) were available. Between projects, the response rate among the implementers varied, ranging from 28.6 to 100%.

### Characteristics of the project leaders and implementers

Most project leaders and prime implementers were female (Supplementary file S1). Some project leaders and implementers were in the early stages of their careers (e.g. 2 years of work experience), while others had extensive work experience (up to 40 years). On average, project leaders had worked for 10.9 years, and implementers for 10.0 years. A majority of the project leaders worked for a municipal government organization. Almost half of the implementers worked at a health organization (e.g. public health service).

### Characteristics of the individual interventions

Most of the individual interventions aimed to increase physical activity or reduce alcohol abuse, and tried to reach youth aged between 4 and 18 years as well as their parents (Supplementary file S2). In the individual interventions, education was the most prevalent strategy, while regulation and citizen participation were least often employed. Almost half of all interventions were implemented in the school or preschool setting, and a majority targeted personal (e.g. knowledge) and social environmental behavioral determinants (e.g. social norms within families). The physical (e.g. availability of playgrounds in the neighborhood), economic (e.g. costs of alcohol) and the political (e.g. legislation regarding the sale of alcohol products in sports cafeterias during youth activities) environmental behavioral determinants were less often targeted.

### Partner involvement in the individual interventions

On average, 4 different partners from 2.68 different sectors were involved during the implementation of an intervention (Supplementary file S2). There was a minority of interventions in which just one partner was involved during implementation. The most frequently involved partners were from the municipal government (i.e. primarily employees from the departments of public health, education and welfare), and from the education, public health and primary care sector. Partners from the secondary care sector and bars and restaurants and other businesses were least often involved. Approximately 60% of the interventions involved no private or citizen partners.

### Composition of the intervention packages

The intervention packages that were implemented each included between 1 and 36 different interventions, with an average of about 17 interventions (Table 1). Nutrition, physical activity and alcohol were more often themes in intervention packages than drugs and smoking. The number of intervention strategies varied between 1 and 5, and on average 3.45 different strategies were employed in the packages. In the intervention packages, education, facilitation and case finding were most frequently employed, while regulation and citizen participation were employed less often. The number of different settings in which packages were implemented ranged from 1 to 7, with an average of 4.45. Most packages included interventions that were implemented in school or preschool settings, outdoor public sites, health or welfare buildings and public buildings. Interventions were less often implemented at home and in commercial buildings (e.g. supermarkets). The packages targeted an average of 3.48 different behavioral determinants, the numbers ranging between 2 and 5. None of the packages targeted just one determinant. The most commonly targeted behavioral determinants were personal factors and factors in the social environment. Factors in the economic and political environments were least often targeted.

**Table 1:** Intervention packages: characteristics and composition, and partners involvement and diversity during the adoption and implementation phases (*N* = 31)

	*N*	Percentage or mean/median (SD) [range]
Characteristics of the intervention packages		
Mean number of interventions implemented		16.8/14.0 (11.1) [1–36]
Theme^a^ (%)		
Nutrition	19	61.3
Physical activity	20	64.5
Alcohol	18	58.1
Drugs	14	45.2
Smoking	15	48.4
Target group^a^ (%)		
Age groups		
0–4 years	9	29.0
4–12 years (primary school)	24	77.4
13–18 years (secondary school)	26	83.9
Adults	21	67.7
Specific groups		
Parents	27	87.1
Low socio-economic status	15	48.4
Ethnic groups	13	41.9
Pregnant women	4	12.9
Mean number of strategies [range 1–5]		3.45/4.00 (1.26) [1–5]
Strategy^a^ (%)		
Education	30	90.9
Regulation	16	51.6
Facilitation	23	74.2
Citizen participation	13	41.9
Case finding	25	80.6
Mean number of settings [range 1–7]		4.45/5.00 (1.71) [1–7]
Setting^a^ (%)		
School/preschool	26	78.8
Sports facility	18	58.1
Outdoor public site	23	74.2
At home	13	41.9
Health or welfare building	22	71.0
Public building	23	74.2
Commercial building	13	41.9
Mean number of targeted behavioral determinants [range 1–5]		3.48/4.00 (0.90) [2–5]
Targeted behavioral determinants^a^ (%)		
Personal	31	100.0
Social environment	29	93.5
Physical environment	22	71.0
Political environment	13	41.9
Economic environment	13	43.3
Partners involvement and diversity during the adoption phase		
Mean number of partners		11.6/10.0 (5.7) [5–27]
Mean number of different sectors [range 1–11]		6.1/6.0 (1.7) [4–10]
Sectors^a^ (%)		
Municipal government	27	87.1
Education	19	61.3
Sports	15	48.4
Welfare	26	83.9
Public health	26	83.9
Primary care	26	83.9
Secondary care	8	25.8
Cultural/recreational sector/social affairs	13	41.9
Transportation and safety	14	45.2
Bars and restaurants	7	22.6
Other businesses	8	25.8
Private partner involved (%)	22	71.0
Mean % of private partners among the total number of partners involved (*N*=24)		17.3/16.7 (14.6) [0–55.6]
Citizens involved (%)	21	67.7
Mean % of citizen partners among the total number of partners involved (*N*=22)		11.0/10.5 (10.3) [0–36.4]
Partners involvement and diversity during the implementation phase		
Mean number of partners		18.7/17.0 (10.9) [5–42]
Mean number of different sectors [range 1–11]		6.9/7.0 (2.1) [3–10]
Sectors^a^ (%)		
Municipal government organization	26	83.9
Education	24	77.4
Sports	19	61.3
Welfare	28	90.3
Public health	25	80.6
Primary care	29	93.5
Secondary care	11	35.5
Cultural/recreational/social	18	58.1
Transportation and safety	14	45.2
Bars and restaurants	10	32.3
Other businesses	9	29.0
Private partners involved (%)	28	90.3
Mean % of private partners among the total number of partners involved (*N*=28)		21.1/21.7 (12.2) [0–40]
Citizen partners involved (%)	27	87.1
Mean % of citizen partners among the total number of partners involved (*N*=27)		17.2/17.1 (11.7) [0–40]

a More than one answer was allowed.

### Partnership diversity during adoption

An average of approximately 12 different partners from about 6 different sectors was involved when decisions to adopt interventions were made (Table 1). In all projects, at least 5 different partners from at least 4 different sectors were involved during this adoption phase. Partners from the municipal government, welfare, public health, primary care, and education sectors were involved in most projects. Partners from the secondary care sector and bars and restaurants and other businesses were least often involved. A majority of the projects involved private partners and citizens in adoption decisions, but they constituted a small percentage of the total number of partners.

### Partnership diversity during implementation

During the implementation of the packages, an average of about 19 different partners was involved, from about 7 different sectors (Table 1). In this implementation phase, projects involved at least 5 and up to 42 partners, from at least 3 to a maximum of 10 different sectors. Partners from the municipal government organization and the education, welfare, public health and primary care sectors were most often involved, whereas partners from the secondary care sector and bars and restaurants and other businesses were least often involved. Private partners and citizens were involved in the implementation of almost all the packages, but again constituted a small percentage of the total number of partners.

### Associations between partnership diversity and composition of packages

**Fig. 1:**
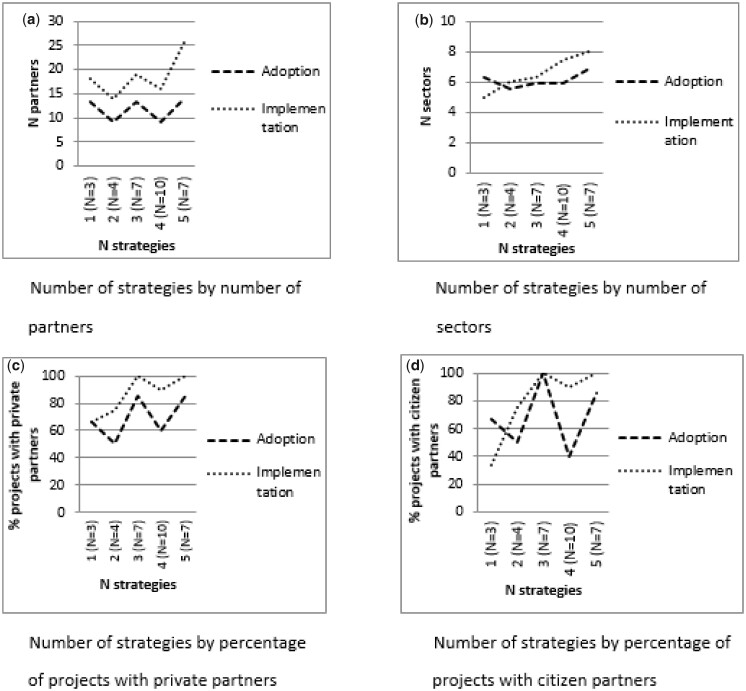
Associations between the number of strategies and partnership diversity during the adoption and implementation phases.

Figure 1a–d shows that partnership diversity during the adoption phase had no clear association with the number of *strategies*. During the implementation phase, the number of strategies tended to be higher if more partners and more sectors were involved and if private partners and citizens were involved.

**Fig. 2:**
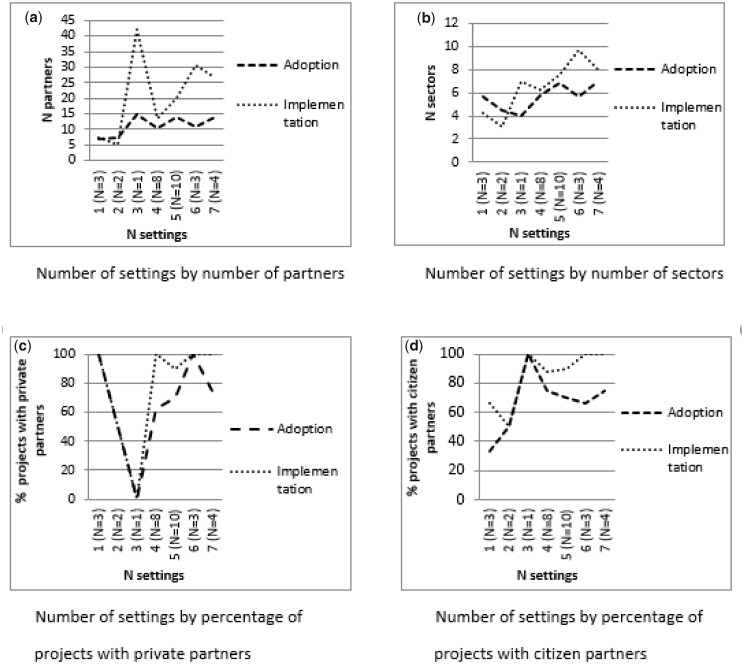
Associations between the number of settings and partnership diversity during the adoption and implementation phases.

Figure 2a–d shows that the number of *settings* tended to be higher if more sectors were involved during the adoption phase. During the implementation phase, the number of settings also tended to be higher if more partners and more sectors were involved, and if private and citizen partners were involved.

**Fig. 3:**
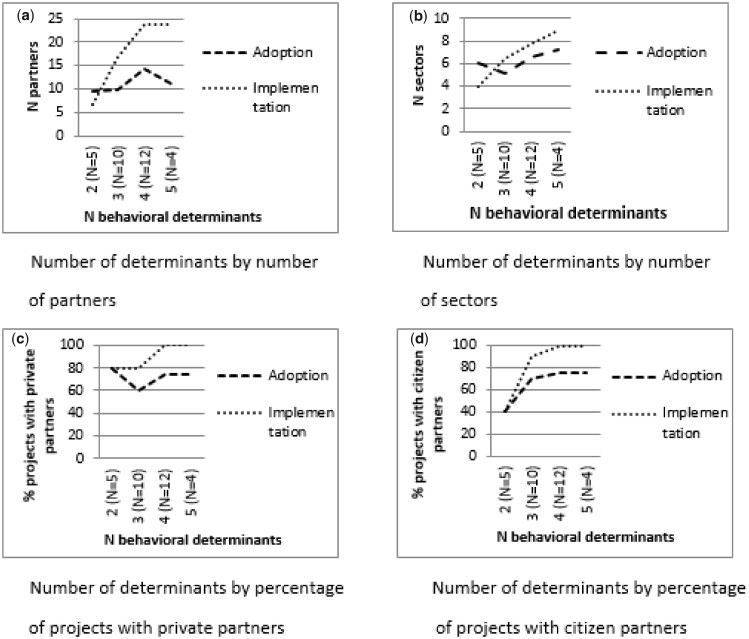
Associations between the number of targeted determinants and partnership diversity during the adoption and implementation phases.

Figure 3a–d shows that the number of *targeted determinants* tended to be higher if more sectors were involved and if citizens were involved during the adoption phase. During the implementation phase, the number of determinants also tended to be higher if more partners and more sectors were involved, and if private partners and citizens were involved.

Finally, there was a positive association between the number of interventions and the numbers of strategies, settings and targeted determinants (Supplementary file S3).

## DISCUSSION

In the context of intersectoral health policymaking, our multiple-case study was guided by the question whether partnership diversity mattered for the composition of integrated HP packages implemented in Dutch municipalities. Almost all projects implemented integrated packages in the sense that they employed several strategies in different settings targeting a variety of behavioral determinants. However, the majority of packages employed particularly education and facilitation strategies in public settings targeting personal determinants. Also, projects primarily included partners from the health, welfare and education sectors, rather than from other policy sectors, private partners and citizens. We found that greater partnership diversity, reflected by more different partners and sectors as well as more private partners and citizens, was associated with more integrated intervention packages, reflected by more diverse strategies, settings and behavioral determinants. This association was especially present during the implementation phase.

Our study is the first to provide quantitative evidence for the association between greater partnership diversity and more integrated intervention packages. Nonetheless, the principal sectors involved (i.e. health, welfare and education), strategies employed (i.e. education and facilitation) and determinants addressed (i.e. personal), still represent a low degree of policy integration according to the typology of intersectoral health policy (Kickbusch, 2010). Although even such a low degree of policy integration may be a noteworthy achievement (Axelsson and Axelsson, 2006; Edvardsson *et al.*, 2012; Varda and Retrum, 2012), two qualitative studies on intersectoral policy found a similar tendency to favor smaller-scale interventions targeting personal determinants over broader policies targeting structural (i.e. physial, economical and political) environmental determinants (Clavier *et al.*, 2012; Holt *et al.*, 2017). Similarly, in a review study on the impact of intersectoral action, only a small minority of the partnerships evaluated by the primary studies addressed structural determinant of health (Ndumbe-Eyoh and Moffat, 2013). We argue that this more common pattern implies that the association we found between partnership diversity and integration of HP packages is not an unconditional one. Although of importance, partnership diversity by itself was found to be insufficient for establishing an approach that targets the full variety of environmental determinants of health behavior (Peters *et al.*, 2017b).

A first condition that may facilitate diversely composed partnerships to implement integrated HP packages, is ‘framing’. For example, one study showed (Holt *et al.*, 2017), that framing health as a means to achieve the objectives of non-health sectors supported the introduction of healthier practices into various settings (i.e. a ‘passive’ setting approach; Whitelaw *et al.*, 2001) rather than policies targeting the health-affecting features of these settings (i.e. a ‘structural’ setting approach; Whitelaw *et al.*, 2001). Therefore, it has been questioned whether putting health—or health behavior, as was the case for the Dutch program—at the center is the best approach to intersectoral policymaking (Breton, 2016; Holt *et al.*, 2017). More than that, it has been suggested that starting with the health argument may be counterproductive (Carlisle, 2010; De Leeuw, 2017; Strøm Synnevåg *et al.*, 2018), and that avoiding ‘the “H” word’ altogether would provide better opportunities to involve non-health sectors in intersectoral partnerships (Howard and Gunther, 2012), p. 35; (De Leeuw, 2017)]. To facilitate structural environmental determinants underlying health and health behavior to become the objects of intervention, a more promising approach could be to make clear how the non-health sectors’ core operations (e.g. ensuring optimal educational opportunities; maximizing anti-poverty measures) contribute to health (Hendriks *et al.*, 2015; Pinto *et al.*, 2015; Holt *et al.*, 2017). As support for such an approach, an early analysis of the policy plans of the projects in the current study concluded that a less central role for the health sector, and formulating broad policy goals, provided better opportunities for higher levels of policy integration in terms of partners involved and strategies employed (Peters *et al.*, 2016). However, encouraging municipalities to frame health problems in line with the structural environmental determinants may require more substantive governmental directions than those provided by a procedural program (Carlisle, 2010; Clavier *et al.*, 2012). Such directions may include predetermining the aims and content of the integrated public health policies and programs to be implemented (Rayner and Howlett, 2009; De Leeuw, 2017).

A second condition that may facilitate diversely composed partnerships to implement integrated HP packages, is ‘management’ (Roussos and Fawcett, 2000; Koelen *et al.*, 2008, 2012). Network management, defined as all deliberate attempts to facilitate or guide interaction processes in a network (Koppenjan and Klijn, 2004) could be a means to support diversely composed partnerships to achieve collective outcomes. Two additional studies on the projects in the present study indeed revealed that partnership diversity was only effective, in terms of implementing integrated HP packages that addressed the full variety of environmental determinants of health, in the presence of intense network management (Peters *et al.*, 2017b; Harting *et al.*, 2019). In diversily composed networks, such management may contribute to collective outcomes by reducing the complexity in the network (e.g. through connecting values and interests) and by creating the active participation and trust needed for non-health sectors to invest in intersectoral health policy (Koppenjan and Klijn, 2004; Varda and Retrum, 2012; Weiss *et al.*, 2016). Apart from managing this ‘policy reality’, project leaders should similarly manage the ‘epidemiological reality’ in order to frame health problems in line with the structural environmental determinants (Peters *et al.*, 2016). Such a complex management task may require highly developed competencies, which perhaps should play a more important role in the education and appointment of project leaders in intersectoral policymaking (De Leeuw and Peters, 2015). Such competencies may include awareness of what boundaries between sectors imply for public health action as well as boundary spanning skills to encourage collaborations across these sectors (Williams, 2002; Holt *et al.*, 2018).

Finally, our study showed that partnership diversity mattered less when decisions were made about the composition of the intervention packages than when these packages were implemented in practice. This finding seems to contradict the suggestion that non-involvement in the adoption phase would be a barrier to partners becoming involved in the implementation phase (Provan and Milward, 2001). However, both the adoption and implementation of integrated HP packages in the projects we studied may be considered to have taken place at the level of operationalized program elements rather than at the abstract level of general policy ideas and norms (Rayner and Howlett, 2009). We argued that the low level of policy integration, reflected by the composition of partnerships and intervention packages, may have been induced by the ministerial program’s focus on health behaviors. Hence, to enable municipal projects to develop ideas for—and reach agreement about—the implementation of more integrated intervention packages at the operational program level, the involvement of non-health sectors, private sectors and citizens may be also required at the abstract level of general policy ideas and norms, that is, in the conception of procedural intersectoral policymaking programs.

### Limitations

A first limitation is that the present study took a quantitative approach, without as well collecting in-depth qualitative data on the process of intersectoral health policymaking or on potentially influential contextual municipal characteristics. Important reasons for this approach were the large number of projects in the ministerial program, the great deal of actors in the local partnerships and the great many interventions that were implemented. However, other study components of our longitudinal multiple-case study, cover part of the policymaking process (Peters *et al.*, 2016, 2017b; Harting *et al.*, 2019). We used those findings to help us interpret the findings from the present study.

A second limitation is that we do not know to what extent the municipal projects that participated in the Dutch ministerial program reflect current policy practice in other Dutch municipalities and other countries. Collecting data from municipalities that did not apply for participation in the program, or that applied but were not allowed to participate, exceeded the scope and the resources of our study. However, the patterns we identified in the composition of the partnerships and intervention packages were quite comparable to those identified in other western countries (Clavier *et al.*, 2012; Holt *et al.*, 2017). This may imply that in such countries the association we found between both these conditions may also be quite similar.

A final limitation is that we operationalized integrated intervention packages in terms of the strategies these employed and the determinants these addressed. However, apart from these two conditions, policy integration also includes whether interventions are implemented in assimilation rather than in isolation or fragmented (Rayner and Howlett, 2009; De Leeuw and Peters, 2015). The sometimes uncertain coherence that was visible in the action plans of the projects included in our study (Peters *et al.*, 2016), indicates that collecting in-depth data on this aspect of policy integration would definitely be an important addition for future studies.

## CONCLUSIONS

Our study is the first to provide quantitative empirical evidence for the assumption that partnership diversity matters for the composition of integrated HP packages. Thus, in order to implement integrated HP packages as a means to improve public health, it seems worthwhile to invest in the challenging endeavor of collaborating with many different partners. However, to bring the structural environmental determinants of health behavior within the reach of HP, additional investments will be needed, such as in the framing of health issues and in network management.

## ETHICS APPROVAL AND CONSENT TO PARTICIPATE

The study was exempt from ethical review according to prevailing Dutch standards because the study was conducted among adults, considered to be low risk, participation was voluntary, and completion of the questionnaires was considered to be equivalent to assent (Central Committee on Research Involving Human Subjects, 2014).

## SUPPLEMENTARY MATERIAL

Supplementary material is available at *Health Promotion International* online.

## Supplementary Material

daaa083_Supplementary_Data
