# Supplementary material for: Does partnership diversity in intersectoral policymaking matter for health promoting intervention packages’ composition? A multiple-case study in the Netherlands
Source: Health Promot Int. 2020 Aug 20;36(3):616–29. doi: 10.1093/heapro/daaa083 (PMC8384381; doi:10.1093/heapro/daaa083)
Supplement: daaa083_Supplementary_Data [file daaa083_supplementary_data.zip › Supplementary file 1 - HPI-2019-0.06.R3.docx]

Table of characteristics of the respondents (31 project leaders and 152 implementers from 31 projects)

|  | N | Percentage or mean (SD) |
| --- | --- | --- |
| *Project leaders* |  |  |
| Female (%) | 28 | 90.3 |
| Mean years of work experience |  | 10.9 (8.8) [2-40] |
| Type of organization (%)  Municipal government organization  Regional Public Health Organization  Other^a^ | 18  11  2 | 58.1  35.5  6.5 |
|  |  |  |
|  |  |  |
| *Implementers* |  |  |
| Female (%) | 110 | 72.4 |
| Mean years of working experience (Valid N=143) |  | 10.0 (7.7) [0-35] |
| Type of organization (%)  Municipal government organization  Health organization^b^  Non-health organization^c^  Other^d^ | 21  71  57  3 | 13.8  46.7  37.5  2.0 |

^a^ = university, high school, welfare organization

^b^ = Regional Public Health Services, addiction care organizations, homecare services, dieticians, general practitioners

^c^ = (pre)schools, sports organizations, welfare and citizens organizations, research institutions, libraries, police stations

^d^ = intervention/project organizations, self-employed
