# Supplementary material for: Does partnership diversity in intersectoral policymaking matter for health promoting intervention packages’ composition? A multiple-case study in the Netherlands
Source: Health Promot Int. 2020 Aug 20;36(3):616–29. doi: 10.1093/heapro/daaa083 (PMC8384381; doi:10.1093/heapro/daaa083)
Supplement: daaa083_Supplementary_Data [file daaa083_supplementary_data.zip › Supplementary file 2 - HPI-2019-0.06.R3.docx]

Table of individual interventions: characteristics and partners involvement (N=302)

|  | N | Percentage or  mean (SD) [range] |
| --- | --- | --- |
| *Characteristics of individual interventions*  Theme^*^ (%; N=292)  Nutrition  Physical activity  Alcohol  Drugs  Smoking | 100  124  120  48  32 | 34.2  42.5  41.1  16.5  11.0 |
| Intended target group^*^ (%; N=292)  Age groups:  0-4 years  4-12 years (primary school)  13-18 years (secondary school)  Adults  Specific groups:  Parents  Low socio-economic status  Ethnic groups  Pregnant women | 15  122  101  59  111  44  27  5 | 5.1  41.8  34.6  20.2  38.0  15.1  9.2  1.7 |
| Strategy^*^ (%; N=292)  Education  Regulation  Facilitation  Citizen participation  Case finding | 210  37  87  22  49 | 71.9  12.7  29.8  7.5  16.8 |
| Setting^*^ (%; N=289)  School/Preschool  Sports facility  Outdoor public site  At home  Health or welfare building  Public building  Commercial building | 116  47  57  27  42  67  34 | 40.1  16.3  19.7  9.3  14.5  23.2  11.8 |
| Targeted behavioral determinant^*^ (%; N=291)  Personal  Social environment  Physical environment  Political environment  Economic environment | 255  171  81  38  22 | 87.6  60.0  28.3  13.3  7.7 |
|  |  |  |
| *Partners involvement in implementation*  Two or more partners involved (%; N=288)  Mean number of partners (N=288) | 242 | 84.0  4.0 (3.7) [1-43] |
| Mean number of different sectors (N=286)  Sectors^*^ (%; N=286)  Municipal government organization  Education  Sports  Welfare  Public health  Primary care  Secondary care  Cultural/recreational/social  Transportation and safety  Bars and restaurants  Other businesses | 141  115  78  82  107  117  16  48  35  13  19 | 2.7 (1.5) [1-8]  49.0  39.9  27.1  28.5  37.2  40.6  5.6  16.7  12.2  4.5  6.6 |
| Private partners involved (%; N=288) | 117 | 40.6 |
| Citizens involved (%; N=288) | 113 | 39.2 |

^*^more than one answer was allowed
