# Supplementary material for: Does partnership diversity in intersectoral policymaking matter for health promoting intervention packages’ composition? A multiple-case study in the Netherlands
Source: Health Promot Int. 2020 Aug 20;36(3):616–29. doi: 10.1093/heapro/daaa083 (PMC8384381; doi:10.1093/heapro/daaa083)
Supplement: daaa083_Supplementary_Data [file daaa083_supplementary_data.zip › Supplementary file 3 - HPI-2019-0.06.R3.docx]

Figure 4. Association between the number of interventions and composition of the intervention package.
